# Killing Many Birds With Two Stones: Hypoxia and Fibrosis Can Generate Ectopic Beats in a Human Ventricular Model

**DOI:** 10.3389/fphys.2018.00764

**Published:** 2018-06-22

**Authors:** Rafael Sachetto, Sergio Alonso, Rodrigo Weber dos Santos

**Affiliations:** ^1^Department of Computer Science, Universidade Federal de São João del-Rei, São João del-Rei, Brazil; ^2^Graduate Program in Computational Modeling, Universidade Federal de Juiz de Fora, Juiz de Fora, Brazil; ^3^Department of Physics, Universitat Politècnica de Catalunya, Barcelona, Spain

**Keywords:** ectopic beats, fibrosis, hypoxia, cardiac electrophysiology, heart simulations, percolation threshold, micro-reentries

## Abstract

During cardiac diseases many types of anatomical and functional remodeling of cardiac tissue can occur. In this work, we focus on two conditions: hypoxia and fibrosis, which are part of complex pathological modifications that take place in many cardiac diseases (hypertrophic cardiomyopathy, hypertensive heart disease, and recurrent myocardial infarction) and respiratory diseases (obstructive pulmonary disease, obstructive sleep apnea, and cystic fibrosis). Using computational models of cardiac electrophysiology, we evaluate if the interplay between hypoxia and fibrosis is sufficient to trigger cardiac arrhythmia. We study the mechanisms behind the generation of ectopic beats, an arrhythmic trigger also known as premature ventricular contractions (PVCs), in regions with high hypoxia and fibrosis. First, we modify an electrophysiological model of myocytes of the human left ventricle to include the effects of hypoxia. Second, diffuse fibrosis is modeled by randomly replacing cardiac myocytes by non-excitable and non-conducting cells. The Monte Carlo method is used to evaluate the probability of a region to generate ectopic beats with respect to different levels of hypoxia and fibrosis. In addition, we evaluate the minimum size of three-dimensional slabs needed to sustain reentries for different stimulation protocols. The observed mechanism behind the initiation of ectopic beats is unidirectional block, giving rise to sustained micro-reentries inside the region with diffuse fibrosis and hypoxia. In summary, our results suggest that hypoxia and fibrosis are sufficient for the creation of a focal region in the heart that generates PVCs.

## 1. Introduction

Cardiovascular diseases frequently promote life-threatening arrhythmias. For some diseases, studies suggest the existence of anatomical triggers for dangerous arrhythmias (de Bakker et al., 1988; Haïssaguerre et al., 1998; Ng, 2006; Jalife, 2011). These pathological regions would repeatedly re-excite the neighboring cardiac tissue, acting as sources, drivers, or foci of ectopic beats. Any area of the heart other than the sinoatrial node, which originates a heart beat is an ectopic pacemaker. In particular, when the region is in the left ventricle, it is the source of premature ventricular contractions (PVCs) (Boineau and Cox, 1973; Ruberman et al., 1977).

In this work, we use a computational model of the human left ventricle to investigate the generation of ectopic beats in a heterogeneous cardiac region under hypoxia and with diffuse fibrosis. Hypoxia accounts for the lack of blood supply for the cardiac tissue, whereas fibrosis is related to the increase of collagen and fibroblast as a reparative or reactive process. Hypoxia can significantly change cardiac electrophysiology and fibrotic regions are non-conducting and non-exciting areas that increase the conduction heterogeneity of cardiac tissue.

The number, diversity and types of processes in cardiac diseases that result on functional or anatomical modifications can be overwhelming, as well as the complexity of their multifaceted interactions. Nevertheless, these two processes, hypoxia and fibrosis, are common in many different cardiac diseases and have been suggested to be related to cardiac arrhythmia. For instance, there is a variety of diseases that can compromise the micro-structure of cardiac tissue in the ventricles via the process of fibrosis. This is the case in hypertrophic cardiomyopathy (HCM) (Maron, 2002), hypertensive heart disease (HHD) (Diamond and Phillips, 2005), and myocardial infarction (MI) (Schmidt et al., 2007). In turn, these diseases, all with the substrate of fibrosis, can also, due to different reasons, induce hypoxia or acute ischemia, and trigger fatal arrhythmias.

Hypertrophic cardiomyopathy is the most common cause of sudden death in young athletes. It is characterized by cardiac and myocyte hypertrophy, myocyte disarray, and fibrosis. Sudden cardiac death may be the first manifestation of the disease. Approximately 70% of all patients with HCM die suddenly (Maron et al., 1995; Ho et al., 2010; Namboodiri and Francis, 2010; Alkon et al., 2012). The high arrhythmic propensity in HCM is due to the combination of the abnormal substrate, like fibrosis, and ischemia or hypoxia (Alkon et al., 2012), in general associated with intense physical exercise. Extreme physical exercise may induce, in HCM patients, diastolic pressure and volume overload of the ventricles, and hypoxemia, i.e., low oxygen saturation in the blood. These can result in a transitory ischemia, usually near the endocardial region. Ventricular arrhythmias related with exercise are strongly correlated to cardiac fibrosis in patients with hypertrophic cardiomyopathy (van Rijsingen et al., 2011). The arrhythmias originate from areas with a high extent of fibrosis or from regions directly adjacent to these areas.

Hypertensive heart disease comprises of structural, functional, and endothelial processes that alter coronary hemodynamics and ventricular function. As in HCM, ventricular enlargement or hypertrophy is also combined with fibrosis. In addition, changes in coronary arterial flow involve ventricular wall compression, luminal obstruction, the increased wall thickening of the hypertensive arteriole and reduced ventricular wall vascularity (Frohlich, 2001). These pathophysiological changes are frequently associated with ischemic heart disease and the combination with the fibrotic substrate increases the risk of sudden cardiac death (Frohlich, 2000).

Most of myocardial infarction (MI) involves the occlusion of a coronary artery of the ventricle or other types of coronary artery diseases. In the acute phase, ischemia and necrosis (Chiong et al., 2011) appear. Early revascularization and the use of new medicaments greatly contribute to improve survival to acute MI. During the healing phase of MI, fibrosis substitutes necrotic cells (Shi et al., 2017). However, post-MI patients remain at substantial risk for ventricular arrhythmias (Pouleur et al., 2010). In fact, most of the deaths in post-MI patients are due to arrhythmia and recurrent MI, i.e., a second episode of acute MI at the same region of the first one (Ørn et al., 2005). Therefore, once again, we recognize the existence of a heterogeneous substrate that involves fibrosis from the first MI episode, hypoxia and ischemia during the recurrent MI, and the relation to fatal arrhythmias.

The aforementioned cardiac diseases suggest a sequence of events: structural changes that involve fibrosis are followed by hypoxia or ischemia, that in turn results in cardiac arrhythmia. However, the relation between fibrosis and hypoxia is not trivial. For instance, some respiratory diseases that involve chronic, transitory, or intermittent hypoxia induce the process of fibrosis in cardiac tissue, and increase the occurrence of cardiac arrhythmias. Studies in patients with chronic obstructive pulmonary disease (COPD), obstructive sleep apnea (OSA), and with cystic fibrosis (CF) have shown that low oxygen saturation during exercise may predispose them to cardiac ventricular arrhythmias (Cheong et al., 1990; Ruf and Hebestreit, 2009; Cintra et al., 2010). Cardiac arrhythmia has been also reported in patients with sleep apnea syndrome (SAS) (Guilleminault et al., 1983). In turn, chronic hypoxia induces cardiomyocyte hypertrophy and interstitial fibrosis in the LV myocardium (Miwa and Sasaguri, 2007; Yamashita et al., 2007). In fact, cardiac fibrosis can develop from different stimuli, including ischemia, inflammation, pressure overload and volume overload. A common feature of all these stimuli is tissue hypoxia, either directly or indirectly, due to increase of oxygen consumption by infiltrating inflammatory cells and activated resident cells (Gao et al., 2014). This process is referred as hypoxia-induced fibrosis and has been a recent important topic of research (Darby and Hewitson, 2016). During chronic hypoxia and pathological repair, the hypoxia pathway might be responsible for driving the process of fibrosis (Watson et al., 2013; Shi et al., 2017).

In summary, hypoxia and fibrosis in the heart appear in many cardiovascular diseases, HCM, HHD, Recurrent MI, and respiratory diseases, COPD, OSA, CF, SAS. In addition, all these diseases are related to cardiac arrhythmias. In this paper, we use a computational model to investigate if the combination of these two pathological processes, hypoxia and fibrosis, is sufficient for the genesis of ectopic beats, a known trigger for life-threatening arrhythmias.

We modified an electrophysiological model of myocytes of the left ventricle (Ten Tusscher and Panfilov, 2006) to include the effects of hypoxia as previously presented in Shaw and Rudy (1997). Diffuse fibrosis is modeled by randomly replacing cardiac myocytes by non-excitable and non-conducting cells. This approach has been employed in a large number of scientific studies to model fibrosis in ventricular (Ten Tusscher and Panfilov, 2007; McDowell et al., 2011; Kazbanov et al., 2016) and atrial (Cherry et al., 2007; McDowell et al., 2015; Alonso et al., 2016) tissues. This approach is justified due to: the lack of a non-invasive technique that can capture the 3D micro-structure of a region with fibrosis; and the complex, distinct and almost unpredictable patterns of fibrosis as identified by histological images (Boineau and Cox, 1973; Campos et al., 2012). As we include this random feature in our simulations, the Monte Carlo method is used to calculate the probability of an injured region to behave as an ectopic pacemaker, assuming that two values characterize the injured tissue: the percentage of fibrosis and the degree of hypoxia.

The results of our simulations suggest that injured regions with both hypoxia and fibrosis generate ectopic beats. Simulations considering only one of these two processes, i.e., only hypoxia or only fibrosis, do not show ectopic beats. The mechanism behind the generation of ectopic beats is unidirectional block, which triggers sustained micro-reentries inside the region with diffuse fibrosis and hypoxia. The analysis of the probability distributions reveals that the simulations that generate ectopic beats have a percentage of fibrosis between 37 and 65%. In addition, the highest probability of ectopic beat formation is obtained for percentages of fibrosis near the percolation threshold, a pure topological metric. This is in agreement with previous results (Alonso and Bär, 2013; Gouvêa de Barros et al., 2015; Alonso et al., 2016). We also observe that the probability increases with the level of hypoxia, i.e., the scenario with most severe hypoxia is the one with highest probability of generation of ectopic beats. Finally, we study how reentries and ectopic beats depend on: (1) the shape of the simulated tissue, cubic vs. rectangular; (2) the total size of the tissue; and (3) the protocol to introduce the action potential wave into the injured tissue.

## 2. Materials and methods

### 2.1. Modeling normal and cardiac myocytes during hypoxia

In order to model the cellular dynamics in our computational experiments, we consider the ten Tusscher model (TT3) of human ventricle myocyte electrophysiology (Ten Tusscher and Panfilov, 2006). For the dynamics of the cellular transmembrane potential (*V*) the following ion currents are considered:

(1) $$\begin{array}{ll} C_{m}\frac{\partial V}{\partial t} & =I_{ion}=I_{Na}+I_{K1}+I_{to}+I_{Kr}+I_{Ks}+I_{CaL}+\\ & I_{NaCa}+I_{NaK}+I_{pCa}+I_{pK}+I_{bCa}+I_{bNa}+I_{KATP};\; \end{array}$$

where *C*_*m*_ is the membrane capacitance. Note that *I*_*ion*_ includes currents from ion channels, exchangers and pumps. Almost all currents depend on the transmembrane potential and on gating variables, **η**, of the Hodgkin-Huxley type.

We modified the ten Tusscher et al. model (Ten Tusscher and Panfilov, 2006) to simulate hypoxia by introducing a *K*^+^ current activated by Adenosine triphosphate (ATP) called *I*_*K*(*ATP*)_ and modifying the *P*_*Ca*(*L*)_ conductivity to be also dependent on ATP. This formulation for modeling hypoxia was introduced before in Shaw and Rudy (1997), although similar alternative formulations are also possible (Kazbanov et al., 2014).

Different degrees of Hypoxia can be simulated by changing the values of [*ATP*_*i*_] from normal (6 mM) to acute (2 mM). According to Shaw and Rudy (1997), this ranges are likely to occur also during acute ischemia. Table 1 shows the impact of the reduction of [*ATP*_*i*_], due to hypoxia on the properties of Action Potential Duration (APD), velocity of action potential propagation, and wave length. Whereas APD depends on the level of hypoxia, velocity is basically constant, as can be seen in Table 1.

**Table 1:** Impact of the reduction of [*ATP*_*i*_], due to hypoxia, on the properties of APD, velocity of AP propagation, and wave length.

**ATPi (mM)**	**APD (ms)**	**Velocity (cm s^−1^)**	**Wave length (cm)**
2	21	31.4	0.66
3	48	33.3	1.6
4	140	34.0	4.7
5	260	34.2	8.9
6	330	34.2	11.3

### 2.2. Action potential propagation on cardiac tissue

The coupling of cells in three-dimensional tissues is modeled with the monodomain formulation, which is given in terms of the transmembrane potential *V* and the vector of state variables, **η**:

(2) $$\begin{array}{rc} \beta C_{m}\frac{\partial V}{\partial t}+\beta I_{ion}\left(V,\text{η}\right) & =\nabla \cdot \left(\text{σ}\nabla V\right)+I_{stim}, \end{array}$$

(3) $$\begin{array}{r} \frac{\partial \text{η}}{\partial t}=f\left(V,\text{η}\right), \end{array}$$

where β is the surface-volume ratio, *C*_*m*_ is the membrane capacitance, *I*_*ion*_ the total ion current, *I*_*stim*_ is the current due to an external stimulus, and **σ** is the monodomain conductivity tensor. The model is further equipped with appropriate initial conditions and no–flux boundary conditions (**n**·σ∇*V* = 0), i.e., the boundary of the tissue is considered to be isolated.

In order to solve the monodomain equations and simulate the action potential propagation in the heterogeneous tissue we used an efficient parallel cardiac solver described in Sachetto Oliveira et al. (2017). This solver uses the Rush-Larsen method to solve the ODEs associated with the TT3 model and the finite volumes method do solve the partial differential equation (PDE) (Equation 2), and can be configured to disconnect a random percentage ϕ of (100 μ m)^3^ volumes, allowing us to model diffuse fibrosis, as described next. The time step (Δ*t*) was set to 0.02 ms for the numerical solution of both ODEs and PDE.

### 2.3. Model of fibrosis

Different types of fibrosis can be observed depending on its spatial distribution (Nguyen et al., 2014): compact, interstitial, patchy, and diffuse. In this work, we consider diffuse fibrosis, which corresponds to the distribution of fibrotic tissue among myocytes in such a way fibrotic and normal tissues are interleaved, forming a complex maze.

We model diffuse fibrosis by randomly removing active tissue volumes of the size of (100 μm)^3^ creating completely disconnected inert regions. The (100 μm)^3^ is roughly the length of individual cells in the longitudinal direction of the tissue. Such approach has been extensively employed before (Ten Tusscher and Panfilov, 2007; Alonso and Bär, 2013; Kazbanov et al., 2016; Vigmond et al., 2016) to study the formations of ectopic beats and reentries in the tissue. The maze of conducting tissue mixed with fibrosis produces fractionated AP propagation.

Fibrosis is modeled via the parameter **σ** in Equation (2). If the fraction of fibrosis in a particular region is ϕ, we assign **σ** = 0 to a given cell with a probability of ϕ. A cell with **σ** = 0 is considered inactive and disconnected from the surrounding cells.

When the fraction ϕ of fibrosis is high, action potential does not propagate through the tissue. On the other hand, action potential wave propagates when the fraction is small. There are intermediate values of ϕ where these two regimes clash and waves are able to propagate in a certain direction but not in the opposite direction due to source-sink mismatches, giving rise to unidirectional blocks responsible for the breakup of the propagating waves. Such effect depends also on the level of ionic remodeling due to hypoxia in the tissue. As explained before, in order to model the degree of hypoxia we change the value of [*ATP*_*i*_].

### 2.4. Calculation of percolation threshold

**Figure 1:**
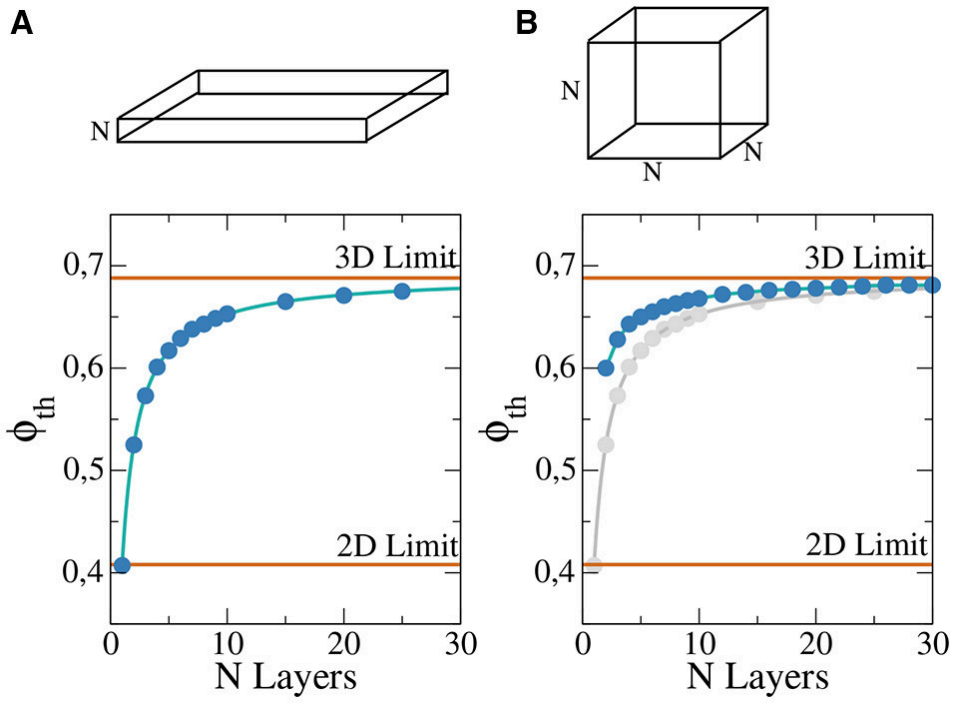
Percolation threshold of a rectangular slab of tissue composed by regular cubic grid of cells for different thickness *N* in a system 400 × 400 × *N* **(A)** and cubic system for different lengths of the cube *N*×*N*×*N* **(B)**. For comparison the analytic limits corresponding to infinite two and three dimensional systems are shown (red lines). Numerical fit (solid blue line) has been calculate to guide the eye and permit to extrapolate to possible higher values of N. For comparison, **(B)** also presents the results of **(A)** in gray.

Waves break in a window of values of the fibrosis fraction in the tissue (ϕ). The window of values is close to the percolation threshold of the grid. Such value corresponds to the maximum fraction of fibrosis at which there is still a path composed of healthy cells connecting one side to the other of the system. Typical theoretical calculations of percolation threshold are done for large systems involving a huge number of cells and therefore, finite sizes effects are discarded. However, a fibrotic region has a finite size and its format or geometry may influence the results. For this reason, we statistically calculate the percolation threshold for the next two types of geometries:

- A rectangular slab of tissue of horizontal lengths 4 × 4 cm^2^ and different thickness: from a single layer (formally a two dimensional grid of cells) to around 25 layers of cells, see Figure 1A.
- A cubic domain formed by a *N*^3^ cells, where we vary the length *N* from 2 cells to around 30, see Figure 1B.

For each pair (*N*, ϕ) we randomly generate 100 different heterogeneous grids and evaluate numerically if the generated models permit the wave propagation from one side to the other. With the results, percolation threshold, ϕ_*th*_, is calculated via linear interpolation. The resulting dependence of ϕ_*th*_ on *N* is presented in Figure 1. As previously noted, for a single layer we approach the two-dimensional limit, see Figure 1A. For slabs with thickness higher than 20 layers the resulting value of ϕ_*th*_ is already close to the theoretical three-dimensional limit value, see Figure 1A. For cubic domains the three-dimensional limit is the same that in the previous case for large systems, see Figure 1B. However, for small system the behavior differs from the two-dimensional limit, see Figure 1B.

## 3. Results

### 3.1. Rectangular slab with a central injured region

**Figure 2:**
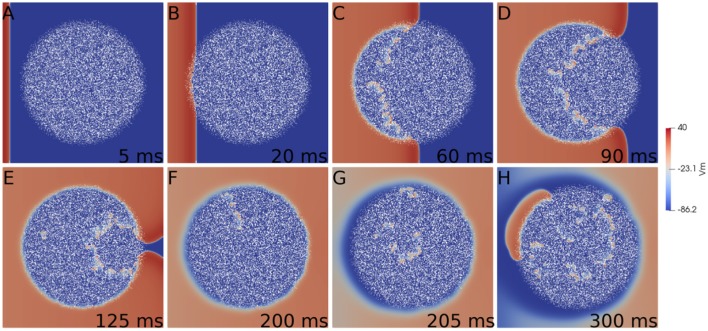
Reentry due to a circular region with fibrosis and hypoxia in the middle of a rectangular slab of cardiac tissue. Different panels show the evolution of the action potential.

First we consider a rectangular slab of healthy tissue with a circular injured region with fibrosis and hypoxia, as shown in Figures 2A–H. If this injured region is small enough, the action potential wave rapidly propagates around, enters from the whole border and excites the whole injured region almost simultaneously. However, if the injured region is large, the wave enters first from the left (from the stimulus side). Since the rest of the border is excited later, propagation in this case is mainly from left to right. If the fraction ϕ of fibrosis is close to the percolation limit of the grid (Alonso and Bär, 2013; Alonso et al., 2016) the waves propagate slower inside the injured region in comparison with the speed on the healthy tissue, see Figures 2C,D. This propagation inside the injured region is highly irregular and continuous break-ups and fusions of waves can occur, see Figures 2C–E. For a certain random combination of cells, the tissue forms source-sink mismatches and unidirectional blocks, i.e., the wave can not propagate from left to right, but can propagate later in the opposite direction (right to left), see Figures 2F,G. The resulting wave in the injured region arrives to the left border and can re-excite the healthy tissue giving rise to an ectopic beat, see Figure 2H. If the leaving wave interacts with a previous wave it can break and generate a rotor. A video of this simulation can be found in the Supplementary Material.

**Figure 3:**
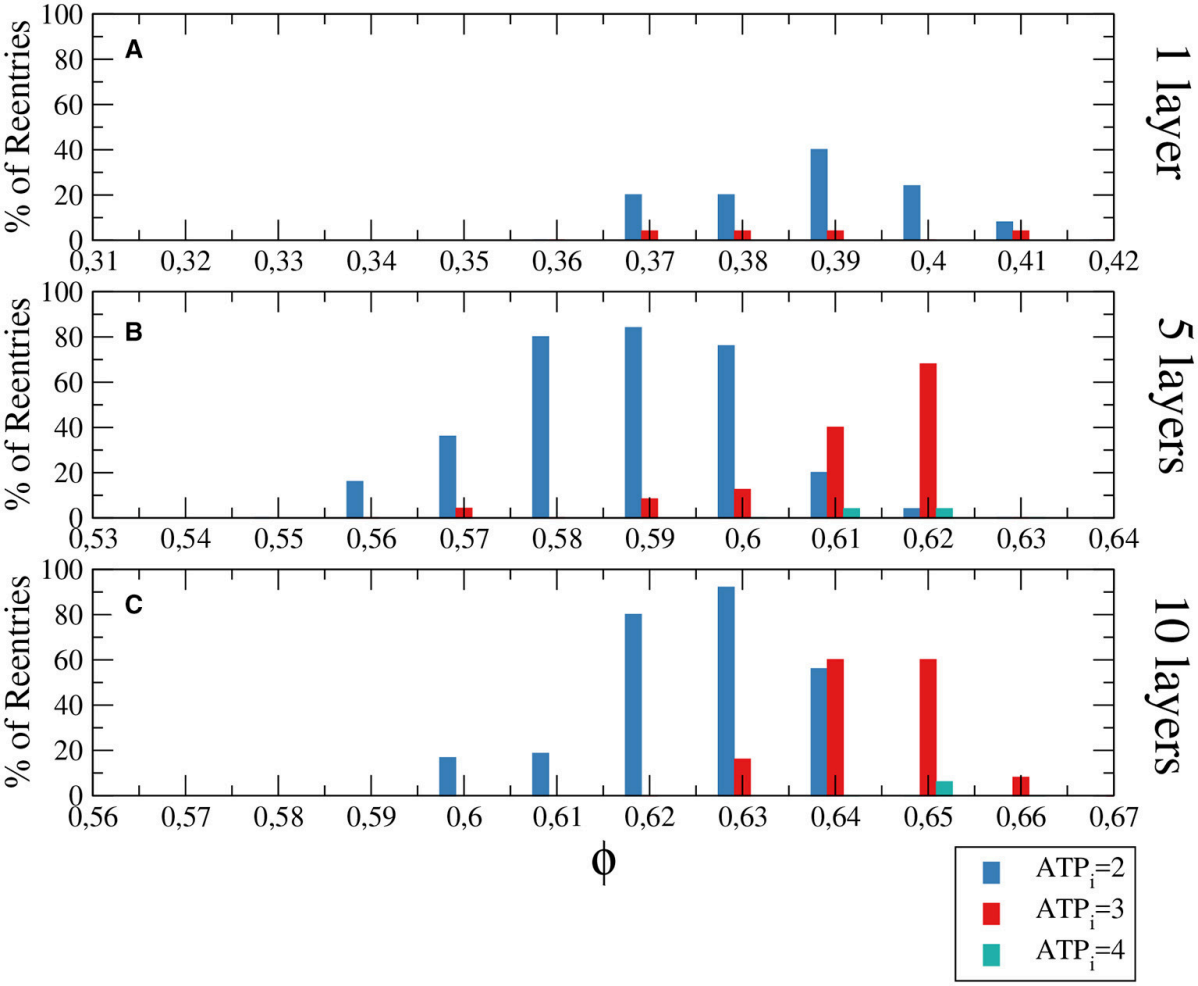
Histograms with the percentage of reentries in a rectangular slab of tissue with an injured circular region with different levels of hypoxia (*ATP*_*i*_) and fibrosis (ϕ). For a two dimensional system corresponding to a tissue with a single layer of cells reentries occurs at low fraction of fibrosis **(A)**, while for 5 layers **(B)** and 10 layers **(C)** the probabilities of reentries increase and reentries occur at higher fractions of fibrosis. The probability to find a reentry decreases for higher values of *ATP*_*i*_. The size of the slab is 4 × 4 cm; The injured region occupies a circular region with radius equal to 1.4 cm.

The generation of reentries depends on different factors, such as the degree of fibrosis and hypoxia. In simulations considering only one of these two processes, i.e., only hypoxia or only fibrosis, ectopic beats are not observed. The fraction of fibrosis of the tissue has to be close to the percolation value of the discrete grid of cells, see Figure 1. Figure 3 shows the probability distribution of reentries as function of the fraction ϕ for different thickness of the slab.

We have analyzed three degrees of hypoxia for different thickness of the slab, see Figure 3. With low degree of hypoxia ([*ATP*_*i*_] = 4mM) reentries are very unlikely to occur and appear for values of ϕ close to the percolation threshold. For severe hypoxia ([*ATP*_*i*_] = 2mM) there is a higher probability of reentries which occur in a wider window of values of the fraction of fibrosis.

### 3.2. Rectangular slab with fibrosis and hypoxia

**Figure 4:**
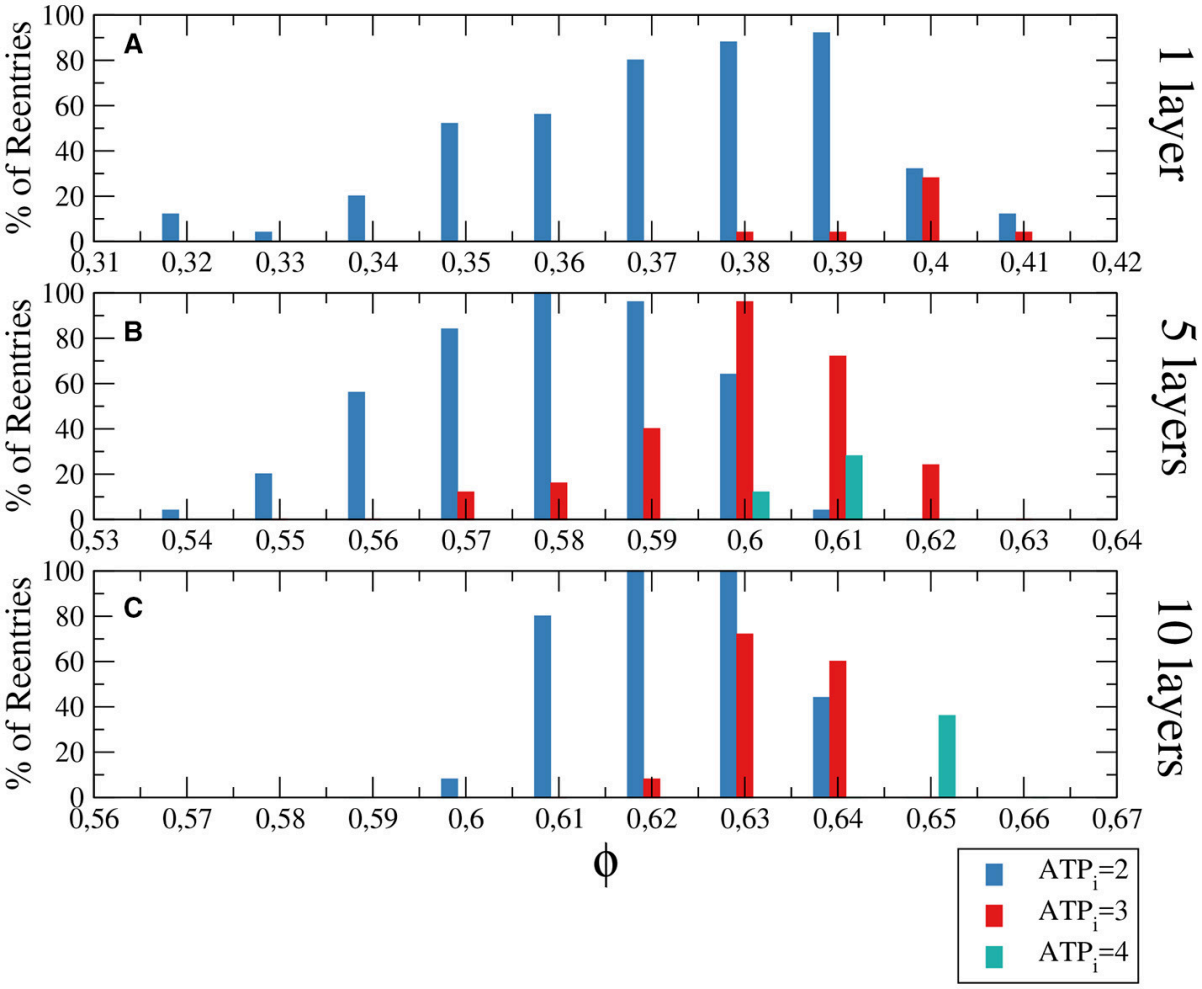
Histograms with the percentage of reentries in a rectangular slab with different levels of hypoxia (*ATP*_*i*_) and fibrosis (ϕ). For a two dimensional system corresponding to a tissue with a single layer of cells reentries occurs at low fraction of fibrosis **(A)**, while for 5 layers **(B)** and 10 layers **(C)** the percentages of reentries are higher and occur at higher fractions of fibrosis. The probability to find a reentry decreases for higher values of *ATP*_*i*_. The size of the slab is 4 × 4 × *N* cm.

The size of the injured region is relevant in the formation of sustained reentries. We perform several simulations using different tissue sizes to demonstrate this relation. First we increase the injured region to cover the whole tissue and a wave is induced at the border of the system for different degrees of fibrosis and hypoxia. The results are shown in Figure 4 and are comparable with the results obtained with a restricted circular injured region shown in Figure 3.

Although the probability of reentries are systematically higher when the whole slab is injured, the range of fibrosis with reentries is basically the same. The probability of reentries increases with the thickness of the slab and, for 10 layers of cells, the probability of reentry for high degree of hypoxia arrives at 100% for several values of ϕ, as we can see in Figure 4C.

### 3.3. Minimum size of a 2D injured rectangular slab that sustains reentries

The difference between the probabilities of reentry obtained in Figure 3, 4 is due to the difference of size of the injured region. Whereas, for example, in Figure 3A the total injured area is 6.16 cm^2^, in Figure 3A the injured area is the whole slab, corresponding to 16 cm^2^.

Next we study the minimum area needed to generate reentries. We use a single layer rectangular slab (2D) and reduce its size systematically. We evaluate the probability of reentries for different values of ϕ keeping [*ATP*_*i*_] = 2mM. The same approach is used in the next sections.

**Figure 5:**
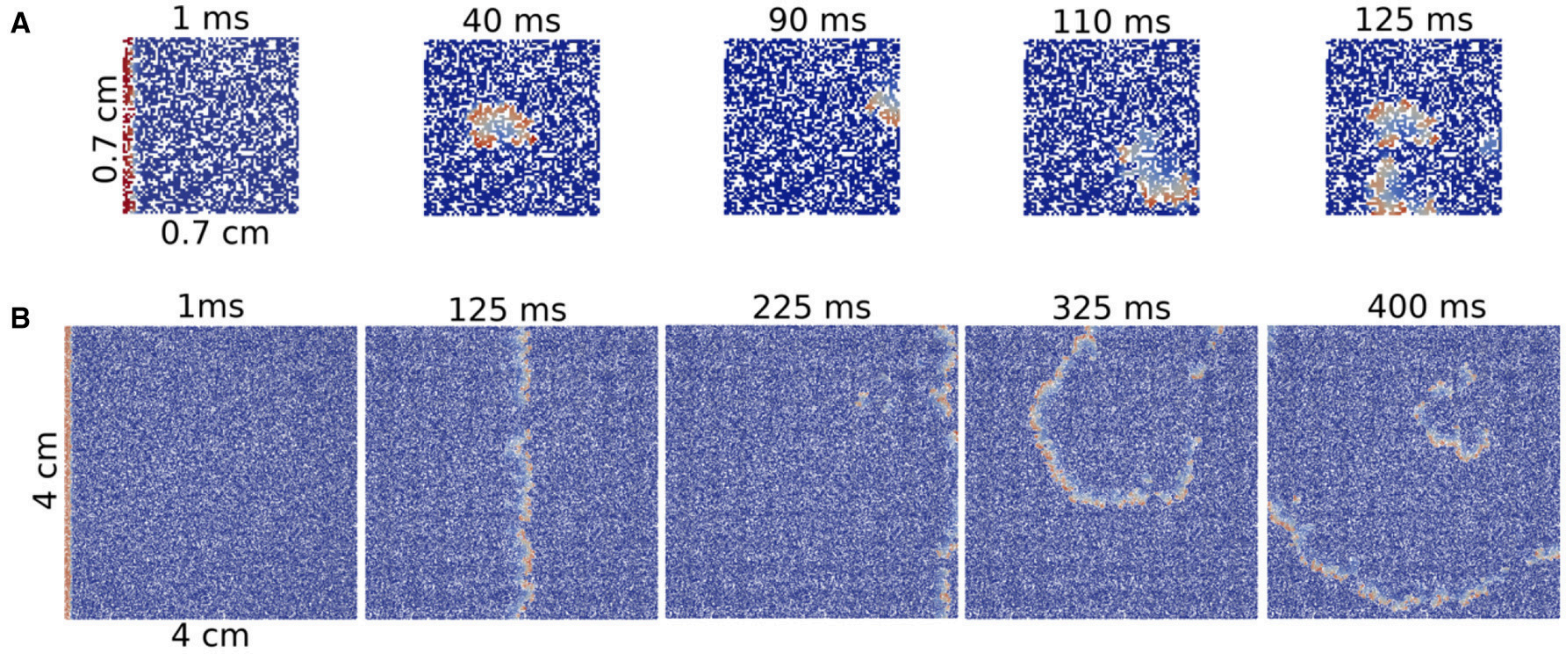
Reentries in different sizes of 2D rectangular slabs of cardiac tissue at different time steps. Reentry obtained in a small system with size 7 × 7 × 0.1 mm **(A)** and reentry obtained in a two-dimensional system with size 40 × 40 × 0.1 mm **(B)**.

We obtain one reentry over one hundred tries for ϕ = 0.38 and ϕ = 0.40 in a system formed by 7 × 7 mm^2^ square (see Figure 5A). However, we did not find any reentry in smaller systems. Note that as our tissue mesh is formed by finite volumes of (100 μm)^3^ , the total number of volumes in such minimum system is 4900.

A comparison between a reentry in the smallest tissue and a typical reentry in a much bigger system is shown in Figure 5. Whereas, for the first case, the excitation can propagate inside the injured region without a clear structure, i.e., in a very fractionated fashion, the reentry shown in the big injured region appears with a well defined shape.

### 3.4. Minimum size of a 3D injured rectangular slab that sustains reentries

One could consider that the total number of finite volumes (FVs) is the most relevant feature that defines the minimum size of the injured region, i.e., a tissue with a high number of injured FVs would have a high probability to generate reentries. To check this hypothesis we perform numerical simulations in a three-dimensional rectangular slab of tissue keeping the thickness constant, 10 layers of cells, and we evaluate the minimum volume under these conditions.

We obtain a single reentry over 25 tries for ϕ = 0.62 in a system formed by a slab with a volume of 7 × 7 × 1 mm^3^. The two-dimensional version of this slab is actually the same case discussed in the previous section. The total number of FVs in such minimum 3D system is 49000, which is 10 times bigger than the number of FVs in 2D. Therefore, these results indicate that the total number of FVs (or myocytes) inside the injured region is not the only relevant parameter for the generation of reentries.

### 3.5. Minimum size of a 3D injured cubic slab that sustains reentries

Next we change the geometry of the domain. We define a cubic injured tissue formed by *L*×*L*×*L* cm^3^, where *L* is the length of the cube. We vary the volume by modifying *L*.

Following the same type of initial condition employed along the previous studies, we systematically reduce the size of the cube to estimate the minimum size which sustains reentries. We find reentries in a cube with *L* = 0.4 cm. In this case the total number of FVs in such minimum system is 64000, which is actually larger than the number of FVs in the previous rectangular three-dimensional slabs (which was 49000).

### 3.6. Dependence on the initial perturbation

**Figure 6:**
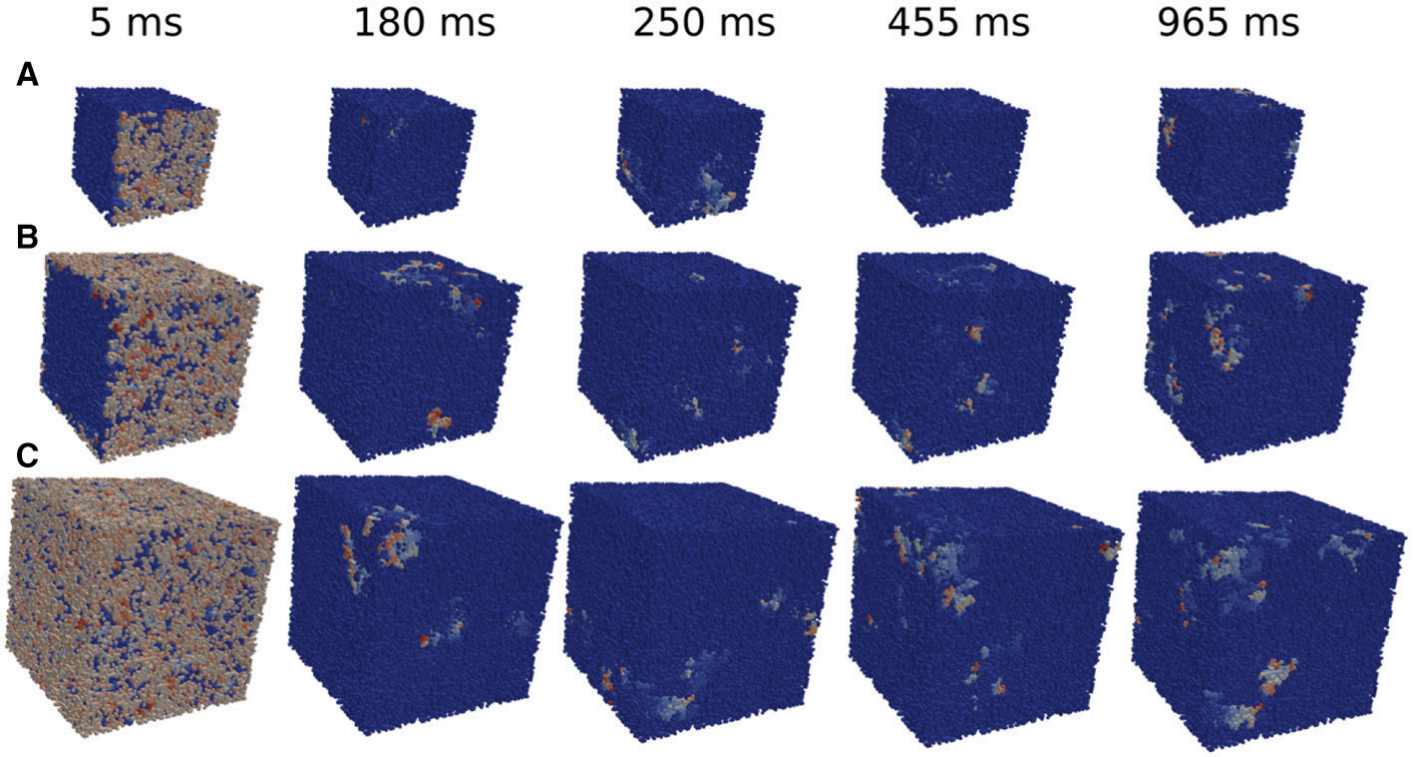
Reentries obtained in cubic grids for different initial protocols and time steps. Activation of a single face of a cubic domain of 0.4 × 0.4 × 0.4 cm **(A)**. Activation of four faces of a cubic domain of 0.6 × 0.6 × 0.6 cm **(B)**. Simultaneous activation of the six faces of a cubic domain of 0.8 × 0.8 × 0.8 cm **(C)**.

The minimum size of a cube to obtain reentries also depends on the initial perturbation. The simulations done previously in the whole tissue slabs are initiated with a perturbation in one of the faces of the volume. However, in the case of the circular injured region in the middle of the slab, see Figure 2 the perturbation from the exterior of the slab continuously trigger the entrance of the wave from different positions. Therefore, the propagation of the waves toward the injured region is different than the oversimplified version shown for example in Figure 5. In order to study how reentries depend on the way AP propagates toward the injured region we implemented and analyzed different initial perturbations, or stimulations, in a cubic domain with fibrosis and hypoxia. See three examples of reentries in Figure 6 with different initial protocols (videos are available in the Supplementary Material).

**Figure 7:**
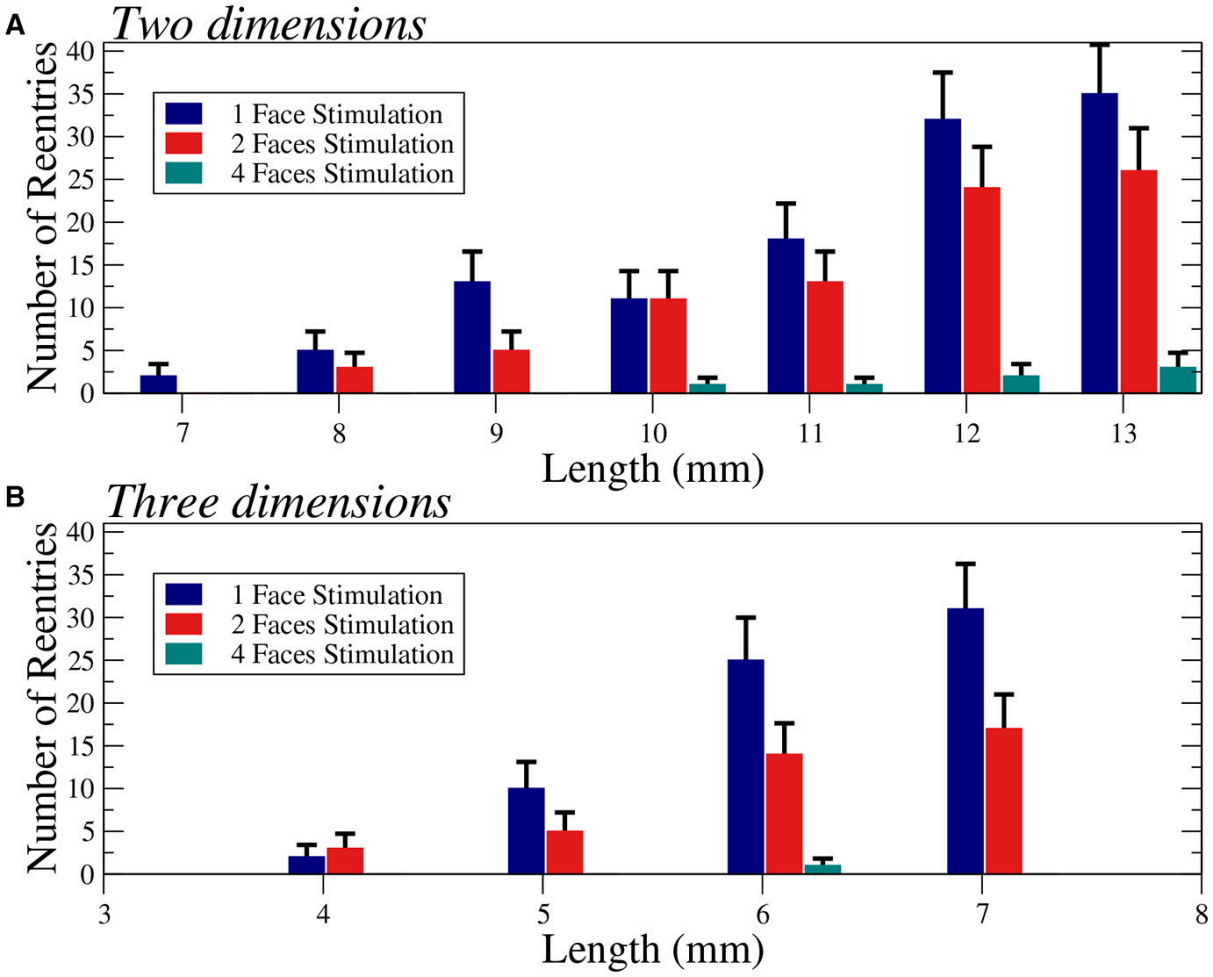
Number of reentries obtained for different sizes of two-dimensional squares **(A)** and three-dimensional cubes **(B)** under different initial perturbation protocols. A total of 600 **(A)** and 300 **(B)** simulations per bar were performed with six different values of the fraction of fibrosis ϕ = 0.35, 0.36. 0.37, 0.38, 0.39, and 0.40 **(A)** and three different values of the fraction of fibrosis ϕ = 0.62, 0.63, 0.64 **(B)**, both with [*ATP*_*i*_] at 2 mM. Error bars are calculated assuming a binomial distribution for the generation of reentries.

We apply several protocols to evaluate the effect of the initial perturbation. We initially perturb a different number of faces of the cubic domain. The most effective way to initiate a reentry inside the cubic domain is the introduction of a perturbation in one face of the cube. Such case is discussed in the previous section, and a particular realization with the minimum size where reentries are observed is shown in Figure 6A. The increase of the size of the injured cube increases the number of reentries (with the same number of statistically independent realizations), as shown in Figure 7. Similar minimum size was obtained when two adjacent faces of the injured cube were perturbed simultaneously. However, the probability to induce a reentry is systematically smaller than the previous case, see Figure 7.

The application of the initial perturbation in four of the faces of the cube synchronizes the excitation inside the injured cube and increases drastically the minimum size needed to obtain reentries. With this stimulus protocol, the minimum size that present reentries increases to *L* = 6 mm, corresponding to a total of 216000 finite volumes. See an example of a reentry in such case in Figure 6B.

Finally, we consider the effect of the simultaneous excitation of all the six faces of the cube and reentries are only obtained with a cube of size *L* = 8 mm, corresponding to a total number of 512000 FVs. See an example of reentry induced by the complete excitation of the six faces of a cube with *L* = 8 mm in Figure 6C.

For the sake of completeness, we have evaluated the changes on the number of reentries for different sizes in two dimensional tissues, see Figure 7A. In two dimensions we observe a similar behavior and for highly synchronized perturbations (four faces) the minimum size is much larger than for less synchronized stimulations (one or two faces).

## 4. Discussion

The computer simulations presented in this work suggest that the combination of fibrosis and hypoxia in a localized region of the myocardium can provide a sufficient condition for the genesis of ectopic beats. The mechanism behind the generation of ectopic beats is unidirectional block inside a region with diffuse fibrosis and hypoxia, where micro-reentries are formed. We note that, although specific regions have been correlated with the generation of ectopic beats, such as the pulmonary veins (Haïssaguerre et al., 1998) during atrial fibrillation or border zone of infarct regions after coronary occlusion (Boineau and Cox, 1973), the mechanism behind this remains unclear. Along decades experiments and numerical simulations have suggested several candidates: abnormal automaticity, triggered activity, such as early or delayed after depolarization, micro-reentries, or even the combination of some of these three (Jalife, 2011). Multiple data available in the literature supports all these mechanisms. Probably, each one might be more specific to a particular disease. However, the results in this work may somehow defy this common sense. Micro-reentries could arise from the combination of hypoxia and fibrosis, two cardiac conditions that appear together in many heart diseases, as hypertrophic cardiomyopathy, hypertensive heart disease, recurrent myocardial infarction, and even in respiratory diseases, such as obstructive pulmonary disease, obstructive sleep apnea and cystic fibrosis. Therefore, our results suggests that a single mechanism could be behind many different pathologies.

It is interesting to note also that among these three mechanism, only micro-reentry, as first proposed to explain PVCs in Boineau and Cox (1973), already suggested that both the electrophysiology as well as the micro-structure of cardiac tissue should be taken into account. Both abnormal automaticity and triggered activity mechanisms focus only on electrophysiology aspects of single myocytes. Nevertheless, recent studies have shown that the presence of fibrosis or other non-conductive or non-excitable cells is of extreme importance in the generation of ectopic beats, even when the altered electrophysiology of myocytes reflects abnormal automaticity or triggered activities (Pumir et al., 2005; Zimik et al., 2015). Therefore, it seems that recent studies are converging toward mechanisms that combine both electrophysiological and micro-structural changes in order to explain the genesis of dangerous ectopic beats.

In this work, we used simple computational models to reproduce hypoxia and diffuse fibrosis. Nevertheless, the result of this combination is far from trivial. First, in the simulations that considered only one of these two processes, i.e., only hypoxia or only fibrosis, ectopic beats were not created. However, even when both conditions were considered, ectopic beats were not always present. This combination does not result in an all-or-none mechanism. The generation of ectopic beats depended on many aspects that were evaluated in this work: percentage of fibrosis, fibrosis pattern, level of hypoxia, size of the injured tissue, its shape, and how AP waves enter the injured region. The way fibrosis is distributed within cardiac tissue was modeled stochastically. Therefore, we used the Monte Carlo method to calculate the probability of an injured tissue to generate ectopic beats.

By varying the percentage of fibrosis and level of hypoxia we observed that the probability of micro-reentries increases with the level of hypoxia, i.e., the scenario with most severe hypoxia was the one with the highest number of reentries. In addition, the simulations that generated ectopic beats had percentages of fibrosis between 37 and 65%, and the highest number of reentries was obtained using percentages of fibrosis near the percolation threshold, a pure topological metric. This is in agreement with our previous results (Alonso and Bär, 2013; Gouvêa de Barros et al., 2015; Alonso et al., 2016).

In these previous studies, we have considered simple and phenomenological models of cardiac myocytes to study the effects of atrial fibrillation (Alonso et al., 2016) or we have considered realistic models of mouse ventricular tissue (Gouvêa de Barros et al., 2015). Here, we consider a human ventricular model (Ten Tusscher and Panfilov, 2006) where hypoxia could be studied at different degrees. The common features of all these studies are the basis of the investigated mechanism of micro-reentry: (1) Diffuse fibrosis generates complex and long reentrant paths within the damaged region; (2) Both action potential and wavelength are short for the cases of mouse AP, remodeled AP due to atrial fibrillation, or, in this paper, remodeled AP due to hypoxia in myocytes of the human ventricle. The combination of long pathways and short wavelengths allows micro-reentry circuits to be formed.

The probability of a compromised region to become an ectopic pacemaker is higher for large damaged regions. We have confirmed this hypothesis with our simulations using both two and three-dimensional systems, see Figure 7. These simulations also show the minimum sizes of the domains where reentries were obtained. The dependence on the size supports the idea that if the system is large, source-sink mismatches are more likely to appear together with long reentrant pathways. Nevertheless, our results suggest that the relation between size and probability of reentry is not trivial and the shape of the injured region and the way AP waves enter it play important roles.

For instance, in Table 2 we summarize our results in terms of minimum required sizes to generate reentries for different tissue geometries and different stimulation protocols. By comparing the simulations that used the same stimulation protocol (stimulus from a single face) we observe that the minimum volume in cm^3^ may vary substantially: 70 × 70 × 1 (0.005 cm^3^), 70 × 70 × 10 (0.05 cm^3^), and 40 × 40 × 40 (0.064 cm^3^). In fact, our results suggest that it is easier to generate an ectopic beat on flat geometries or on thin slabs than on cubics geometries. Coincidentally, the most common infarct is known to be the sub-endocardial one, with a geometry of the damage tissue that can be described as a thin slab near the endocardial surface of the heart. Nevertheless, if we keep the geometry fixed Figure 7 clearly shows that by increasing the volume or size of the injured region sustained reentries are more likely to occur.

**Table 2:** Minimum sizes needed to support reentries using different geometries and different stimulation protocols. FV, Finite Volume.

**Grid**	**Perturbation**	**Geometry (FVs)**	**# FVs**	**Total volume (cm^3^)**
*L*×*L*×1	1 Face	70 × 70 × 1	4900	0.005
*L*×*L*×1	2 Faces	80 × 80 × 1	6400	0.006
*L*×*L*×1	4 Faces	100 × 100 × 1	10000	0.01
*L*×*L*×10	1 Face	70 × 70 × 10	49000	0.05
*L*×*L*×*L*	1 Face	40 × 40 × 40	64000	0.064
*L*×*L*×*L*	4 Faces	60 × 60 × 60	216000	0.216
*L*×*L*×*L*	6 Faces	80 × 80 × 80	512000	0.512

Table 2 also shows how micro-reentries depend on the different possible entrances of the wave into the injured region. We have found that when the borders are well synchronized the minimum injured volume that is needed to generate reentries is much bigger than when the borders are loosely synchronized. For instance, the last three lines of Table 2 show the minimum volumes for different initial conditions when the geometry of the injured region is a cube, see also Figures 6 , 7.

Our results on the minimum size of fibrotic regions can be compared with measurements taken from human ventricles with late-enhancement magnetic resonance imaging (LE-MRI). The studies reported in Wu et al. (1998); Ørn et al. (2007), and de Haan et al. (2011) reported scars of a minimum size around 0.1 cm^3^ for patients with a history of ventricular tachycardia. In our simulations we have found that it is possible to generate an ectopic pacemaker on injured regions with much smaller sizes (one order of magnitude smaller). This discrepancy could be related to the lack of spatial resolution of nowadays LE-MRI exams. Nevertheless, the presented minimum size regions have very low probability to behave as an ectopic pacemaker, see Figures 6 , 7. Therefore, although the occurrence of reentries in such small regions is theoretically possible, it is very unlike to occur.

There are many limitations in this study. First, we have used simple models for both fibrosis and hypoxia. For instance, different types of fibrosis can be observed (Nguyen et al., 2014): compact, interstitial, patchy and diffuse. In this work we have only used a simple model for diffuse fibrosis. In addition, the results and conclusions presented in this work should be taken cautiously. As we have previously mentioned, although hypoxia and fibrosis are found together in many heart and respiratory diseases, they are usually accompanied by many other pathological conditions. Although the complex interactions of pathological conditions other than hypoxia and fibrosis are out of the scope of this work, we list here a few interactions that deserve further investigations in future works. In respiratory diseases hypoxia has been co-observed with oxidative stress (Yin et al., 2012; Ramond et al., 2013; Debevec et al., 2017). It has been shown that the combination of hypoxia and oxidative stress can be quite complex, involve multiple scales of cellular metabolism and lead to further reduction of APD (Zhou et al., 2009). In the case of acute ischemia, whereas hypoxia is present and has been shown to be the condition that most affects AP waveform (Shaw and Rudy, 1997), hyperkalemia and acidosis are also important conditions that can considerably reduce AP wave velocity and, as consequence, further reduce wavelength. Whether the aforementioned conditions could, in theory, corroborate with the described micro-reentry mechanism, and increase the probability of the genesis of ectopic beats, other conditions could counteract it. For instance, during fibrosis a possible connection between myocytes and fibroblasts could increase the APDs of myocytes (Kohl and Gourdie, 2014). However, it is still an open question if fibroblasts are electrically coupled to the myocytes (Kohl and Gourdie, 2014). Therefore, we employ here a simple approach assuming that fibroblasts do not interact electrically with myocytes and they basically act as barriers to the action potential propagation. This approach has been employed in a large number of scientific studies on the modeling of fibrosis in ventricular (Ten Tusscher and Panfilov, 2007; McDowell et al., 2011; Kazbanov et al., 2016) and atrial (Cherry et al., 2007; Alonso et al., 2016; McDowell et al., 2015) tissues. Such approach has been also employed in highly detailed microscopic models of cardiac tissues where cells are disconnected by barriers or by dead cells (Jacquemet and Henriquez, 2009; Hubbard and Henriquez, 2014; Gouvêa de Barros et al., 2015). Other studies consider different approaches to model the electrical interaction between fibroblasts and the myocytes (Xie et al., 2009; Nayak et al., 2017). Finally, we highlight that this is a pure theoretical study, and therefore, further experimental validations are pending.

In summary, the combination of fibrosis with hypoxia in a localized region of the myocardium can provide the sufficient condition for the genesis of ectopic beats and reentries in a human ventricular model. The size, shape and geometry of the injured region, as well as the electrophysiology remodeling due to hypoxia are important features that determine the probability of an affected region to behave as an ectopic pacemaker, a trigger for life-threatening arrhythmias.

## Author contributions

RS, SA, and RdS conceived the numerical experiments. RS conducted the numerical experiments.RS, SA, and RdS analyzed the results. All authors reviewed the manuscript.

### Conflict of interest statement

The authors declare that the research was conducted in the absence of any commercial or financial relationships that could be construed as a potential conflict of interest.
